# Cutaneous Manifestations of COVID-19: An Experience From Oman

**DOI:** 10.7759/cureus.16667

**Published:** 2021-07-27

**Authors:** Aisha Al Ali, Safiya Al-Shidhani, Fatma Al-Balushi, Mohammed Alhinai, Abdul Rahman Al-Azri, Sultan Al Lawati Al Lawati, Farah Al Ghailani, Reham Al Riyami

**Affiliations:** 1 Dermatology, Al-Nahdha Hospital, Muscat, OMN; 2 Internal Medicine, Gastroenterology, Al-Nahdha Hospital, Muscat, OMN; 3 Oral Medicine, Al-Nahdha Hospital, Muscat, OMN; 4 Internal Medicine, Al-Nahdha Hospital, Muscat, OMN

**Keywords:** cutaneous manifestations, sars-cov-2, corona virus, skin manifestations, covid-19

## Abstract

Objectives: To identify the cutaneous manifestations in COVID-19 disease in Oman.

Methods: The study was conducted in two phases with initial cross-sectional data collection with subsequent telemedical investigations of late skin manifestations including confirmed COVID-19 patients evaluated at Al-Nahdha Hospital and local health centers in Muscat from March 22 to June 2, 2020.

Results: The total number of patients included in the study was 374. Cutaneous manifestations were observed in 1.87% (n=7) of patients at presentation with an additional 1.6% (n=5) on follow-up. The types of skin reactions included maculopapular rash (n=6), urticaria (n=2), transient pruritic erythema (n=1), pruritic palmoplantar erythema (n=1), pustular eruption (n=1) and flare-up of atopic dermatitis (n=1).

Conclusions: The low percentage of skin lesions is not contradicting previous data and it might just reflect under-reporting of skin rash in the context of the presence of more severe symptoms in our sample population. Skin lesions can still be utilized to treat patients as suspected cases until proven otherwise as it can be a silent clue in asymptomatic patients.

## Introduction

Since late December 2019, the COVID-19 outbreak has spread from Wuhan, China to the rest of the world causing significant impacts on health systems and the global economy. COVID-19 infection is now well described in terms of its presentation and disease course. The incubation period of COVID-19 is estimated to be around five days [1]. It generally produces non-specific symptoms like body aches, fever and cough that might be associated with a variety of other symptoms like sore throat, shortness of breath, chest pain, nausea, diarrhea and headache among other less frequent symptoms [1,2].

One of the initial studies in Italy reported just above 20% of COVID-19 patients developing skin lesions. The reported skin lesions included erythematous rash, vesicles and generalized urticaria. No specific association with disease stage or severity was reported by the same investigators [3]. Large-scale studies and case reports were subsequently released reporting more extensive illustrations and classification of skin associations that were seen in confirmed or suspected COVID-19 patients with skin rash [4].

It was assumed that these lesions could be induced directly by the virus effect or secondary to associated complications resulting in vascular inflammation and occlusion caused by coagulation abnormalities and vascular damage due to COVID-19 [5-7].

There is limited data on the cutaneous manifestations of COVID-19 and the experiences in the middle east countries in general and in Oman in particular, therefore we report here the outcome of a cross-sectional study that focused on the cutaneous lesions seen in patients who were affected by COVID-19 within two weeks after confirmation of disease.

## Materials and methods

The study was conducted in two phases. The first phase included cross-sectional analysis of COVID-19 patient demographics and clinical presentation with a focus on skin manifestation at the time of diagnosis, followed by a second phase of telemedical investigation of any subsequent late skin presentation.

The sample size for the study was calculated with a 95% confidence level based on the population of all confirmed patients with COVID-19 in Oman up to May 5, 2020. The estimated sample size needed was 373 subjects and the actual sample that was included in the study is 374. Data was extracted from Al Shifa Health Care computer system and recorded electronically using an Excel sheet. Data were analyzed and reported descriptively.

At the first phase, all COVID-19 patients who presented from March 22 to June 2, 2020, as outpatients or admitted as inpatients at Al-Nahdha Hospital, and all outpatients from COVID-19 focal health centers in Muscat Governorate, were included in the study. Patients were included if they had positive severe acute respiratory syndrome coronavirus 2 (SARS-CoV-2) RNA polymerase chain reaction (PCR) test who presented in the period between March 22 to June 2, 2020, and were residents in Muscat Governorate. Patients who had been diagnosed with COVID-19 outside Muscat Governorate were excluded. The data collected included basic demographics, medical background and COVID-19 examination details (Table 1).

**Table 1:** Demographic & clinical characteristics of COVID-19 patients in Muscat

	Male	Female	Subtotal	Grand total
Age Group				
0-19	8 (2%)	11 (3%)	19	374
20-39	109 (29%)	34 (9%)	143	
40-59	119 (32%)	34 (9%)	153	
60+	38 (10%)	21 (6%)	59	
Health institution				
Al Nahdha Hospital	251 (67.2%)	77 (20.4%)	328	374
Health center	23 (6.2%)	23 (6.2%)	46	
Nationality				
Non-Omani	179 (47.8%)	28 (7.5%)	207	374
Omani	95 (25.5%)	72 (19.2%)	167	
Associated comorbidities				
Hypertension	59 (16%)	27 (7%)	86	374
Diabetes mellitus	72 (19.2%)	23 (6.2%)	95	
Asthma	13 (3.5%)	13 (3.5%)	26	
Coronary artery disease	14 (3.7%)	1 (0.3%)	15	
Hyperlipidemia	17 (4.6%)	5 (1.3%)	22	
Kidney disease	6 (1.6%)	4 (1.1%)	10	

In the second phase of the study, the same cohort of patients included initially were further contacted with a specific set of questions to investigate the development of any late skin presentation. A total of 310 out of the initial cohort were followed up, after exclusion of confirmed dead patients, unreachable patients and those who had skin lesions at initial presentation. A telemedicine clinic was set up for screening patients remotely. Patients were informed about the purpose of the telemedical encounter and consent was taken verbally. Briefly, history was taken regarding the development of skin lesions within the following two weeks of confirmed diagnosis of COVID-19 disease. Patients were asked to describe the location, associated symptoms, exposure to any medications and the duration until resolution of the lesions. Furthermore, a pictorial reference was provided to the participants who confirmed the development of skin lesions for a better description. All data were added to the original datasheet of the first phase.

## Results

A total of 374 patients confirmed with COVID-19 disease by using SARS-CoV-2 RNA PCR were enrolled in the study. Fifty-two percent (n=195, 52%) were admitted as inpatients at Al-Nahdha Hospital, 13% (n=48) were intubated and transferred to Intensive Care Units at other tertiary hospitals and 35% (n=131) were managed as outpatients with home-based care and quarantine. The majority of cases included in the study attended Al Nahdha Hospital (n=328, 88%), whereas 12% (n=46) were evaluated at local health centers and managed with home-based care and quarantine. Omani nationals accounted for 45% (n=167) and non-Omani patients accounted for 55% (n=207).

**Figure 1:**
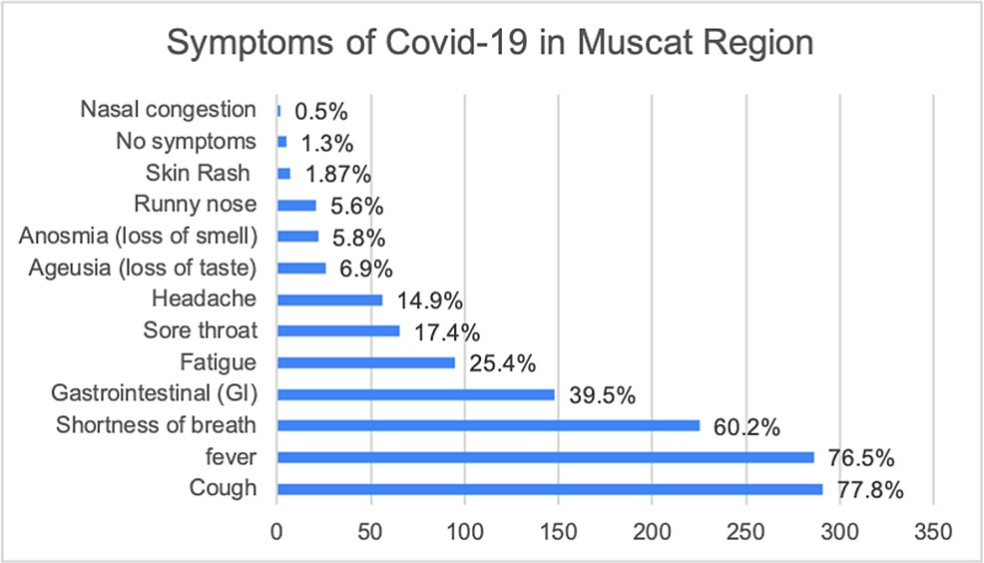
Symptoms of COVID-19 in Muscat Region

The mean age of affected patients was 43.5 years (SD=15.27, age range: 25 days-85 years). There were more males than females [73.3% (n=274) males, 26.7% (n=100) females]. The most affected age group was between 30 and 49 years old with a total of 50.5% (n=189). A total of 248 (66.2%) of patients were younger than 50 years old and 33.7% (n=126) were 50 years old and above. The demographic and pre-existing medical conditions of the sample collected are demonstrated in Table 1. The incidence of clinical features developed following COVID-19 infection in these patients is shown in Figure 1.

**Figure 2:**
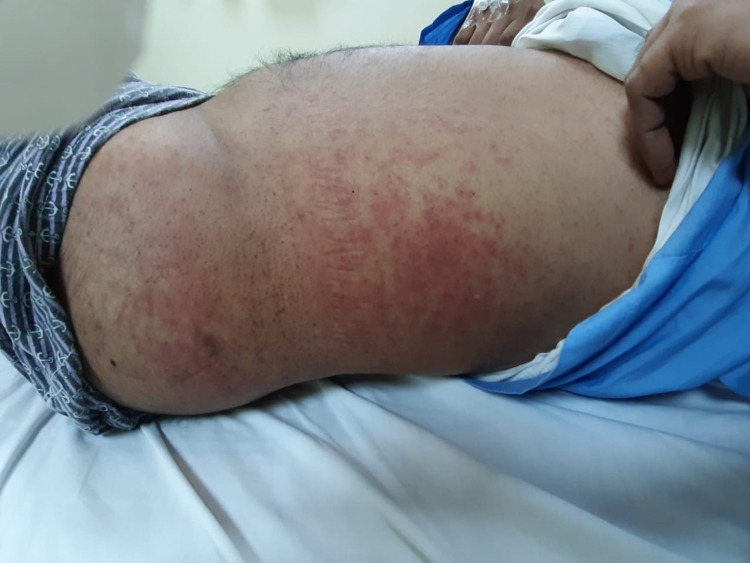
maculo-papular rash

**Figure 3:**
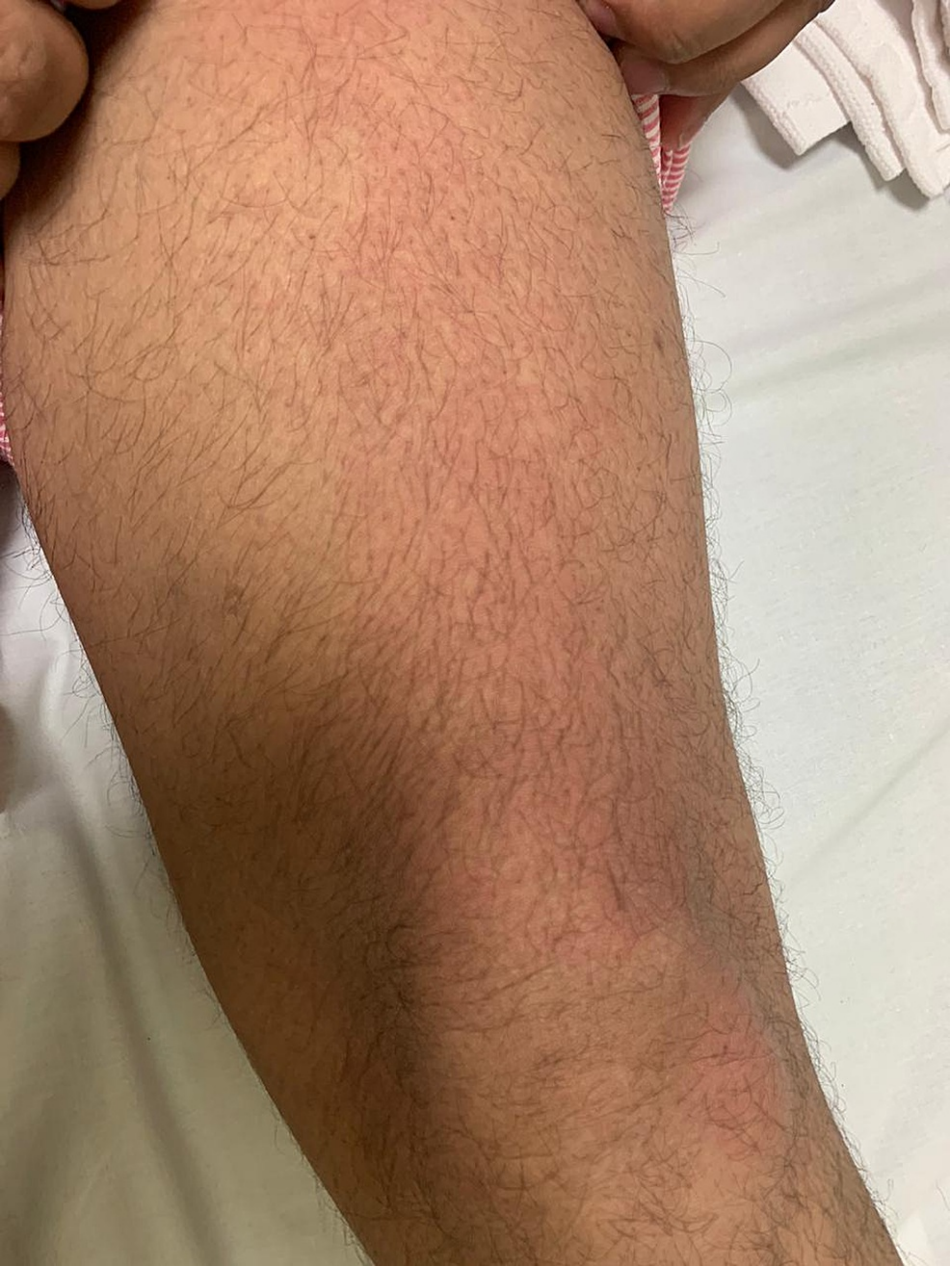
transient pruritic erythema

**Figure 4:**
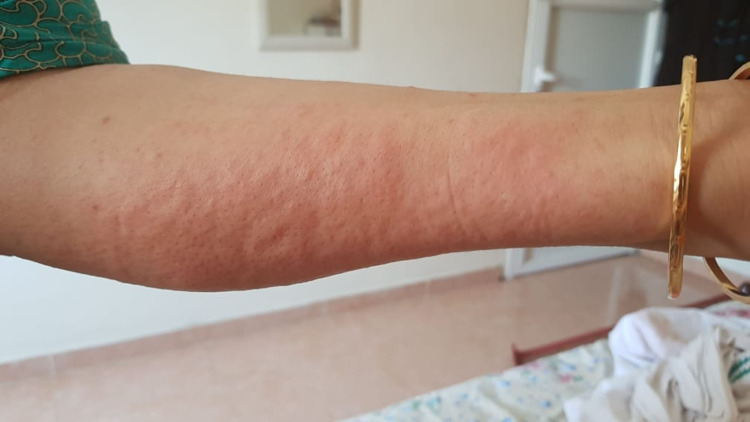
Urticarial Eruption

**Figure 5:**
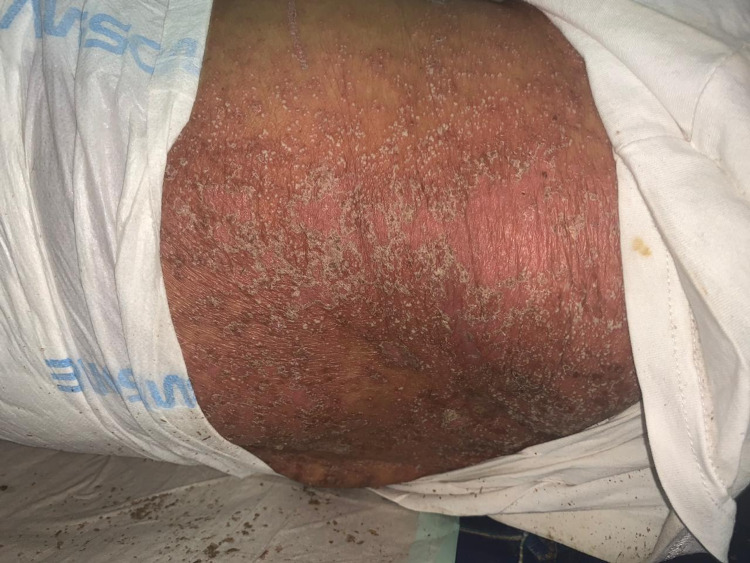
pustular eruption with erythematous background

Skin lesions were documented in 1.87% (n=7) of the 374 patients. The types of skin lesions included maculopapular rash (n=2) (Figure 2), transient pruritic erythema (n=1) (Figure 3), urticaria (n=1) (Figure 4), palmoplantar erythema with itching (n=1) and pustular eruption with erythematous background (n=1) (Figure 5).One patient had a flare-up of pre-existing atopic dermatitis.

The subsequent telemedical follow-up revealed an additional five patients (out of 310 reachable patients) who developed skin rash (1.6%) within two weeks of confirming COVID-19 infection. Four of them had maculopapular rash and one urticarial rash. Skin lesions healed without using topical treatments except in one patient with maculopapular rash. There was preceding history of different drug intake with combinations of antibiotics, hydroxychloroquine and antivirals before the onset of the rash in all of the five patients.

## Discussion

In this study, we described the skin manifestations in COVID-19 inpatients and outpatients who have been evaluated at Al Nahdha Hospital and local health centers in Muscat Governorate in Oman.

The majority of affected patients were middle-aged men. The mean age of included patients was 43.5 years (SD=15.27), which is comparable with what was reported in an international registry of 716 patients from 31 countries [8]. However, the mean age in our study was younger than some previous large reported studies [2,9]. There was an obvious male predominance which is consistent with other published international studies [9,10].

During the time of the study, it was noticed that non-nationals were more commonly affected compared to the Omani population although we included all patients with COVID-19 who were seen during the period from March 22 to June 2 regardless of their nationalities. Several factors have been hypothesized to explain this observation including the ratio of national to non-nationals in the Muscat Governorate region, possibly less optimal lifestyle as some are living in groups, crowded rooms and campuses with possible financial barriers [11].

In our study, the associated chronic medical conditions were seen less frequently than other reported studies [2,9]. Diabetes mellitus was the most common co-morbidity followed by hypertension, asthma and dyslipidemia. Most of the patients had cough and fever as presenting symptoms followed by shortness of breath which is consistent with previous reports [11-13].

Skin lesions were seen in 1.87% at initial presentation of all affected patients and an additional 1.6% developed skin lesions within the following two weeks. This is smaller than most of the reported studies, with the highest reported incidence of 20.4% of all COVID-19 patients from the first prospective study in Italy [3]. Although, a very low incidence of only 0.2% was reported in one of the initial Chinese studies of 1099 positive cases [14]. In the first phase of the study, skin lesions presented variably as maculopapular rash, transient pruritic erythema, urticaria, palmoplantar erythema with itching, pustular eruption with erythematous background and flare-up of pre-existing atopic dermatitis. Patients who developed skin lesions had them mainly along with other symptoms of COVID-19 before the initiation of any treatment and these included the maculopapular eruption, transient pruritic erythema, palmoplantar pruritic erythema and flare-up of pre-existing atopic dermatitis. Some patients developed pustular eruption with erythematous background and urticarial rash that presented after initiation of treatment.

In our study, the additional five patients who noticed skin lesions within two weeks of infection had a preceding history of drug intake before the onset of the rash. Four of them had maculopapular eruption and one had urticarial wheels. The total accumulative number of patients with skin lesions from our study was 12. The number of patients who developed different skin lesions is illustrated in Table 2.

**Table 2:** Types of skin lesions reported by COVID-19 patients

Type of lesion	Number of patients
atopic dermatitis flare-up	1
erythematous rash (maculopapular rash)	6
palmoplantar erythema with itching	1
pustular eruption with erythema (AGEP) drug eruption	1
transient pruritic erythematous patch on thighs, lasted 48 hours	1
urticaria	2
Total	12

The maculopapular rash was the most common skin lesion observed in confirmed COVID-19 cases followed by urticaria in our study, and this is consistent with what was reported in the literature [3,4,8]. These two skin eruptions are non-specific findings and can present with many other viral infections or as an adverse reaction to drugs. Since these symptoms are not specific, they might not be helpful for early diagnosis of COVID-19 disease particularly. They may, however, raise the suspicion of any viral illness including COVID-19 infection.

The transient pruritic erythema on extremities and trunk and the palmoplantar pruritic erythema were noticed at the same time as other symptoms and resolved with symptomatic treatment. These manifestations might be more potentially associated with COVID-19 disease but there was no clear reported data with similar presentations until recently, where palmoplantar involvement was frequently found and reported [15]. One patient had a severe flare-up of pre-existing atopic dermatitis that required high doses of systemic steroids.

The last case of skin reaction with pustular eruption is more likely to be a drug reaction, known as acute generalized exanthematous pustulosis (AGEP), triggered by ceftriaxone that was part of the patient’s regimen treatment. The diagnosis of AGEP was clinical and the lesions resolved gradually after discontinuation of this medication along with symptomatic treatment. The improvement of the patient's condition after stopping the offending drug supported the diagnosis of AGEP. The prescription of multiple drugs for COVID-19 patients increases the likelihood of developing adverse drug reactions and this should be considered as a possible cause for developing skin lesions.

Many other skin lesions have been reported in other studies but were not seen in our sample population. In one study, among skin lesions reported in confirmed and suspected cases of COVID-19, acral lesions were unexpectedly common, presenting in 51% of patients with chilblain-like lesions being the most frequent presentation [16]. This was also reported in a recent study where a high percentage of chilblain-like acral pattern was observed in 24.6% of the population included in the study [17].

It was explained in several studies that chilblain-like lesions could be linked with COVID-19 given that these lesions were observed during warm weather which is against the usual natural history for development of chilblain, increasing incidence observed by dermatologists during that period and the presence of history of contact with COVID-19 cases. However, in most cases, chilblain was noticed later in the disease course which was hypothesized to be related to the microthrombi which is expected due to the deranged coagulation profile noticed in severe COVID-19 patients [4,16,18]. It has been observed in a recent study that pernio/chilblain were recurrent in the sample population with subsequent cold exposure [19].

Some of the skin manifestations reported in other studies suggest the possibility of vascular injury, inflammation and thrombotic vasculopathy as potential underlying pathophysiology. Those manifested as petechial rash, Kawasaki disease in pediatric and adult patients, livedo reticularis, livedo racemose, acral ischemia and retiform purpura [5-7,20-23]. In addition, pityriasis rosea-like lesions were reported as an associated skin rash in confirmed cases of COVID-19 disease [24]. These less common presentations were not encountered in our study. This might be attributed to different factors, including different genetic background, younger population with fewer co-morbidities and different genomes of SARS-CoV-2. Furthermore, it can be cautiously suggested that skin lesions might be underreported by patients and physicians as vigilance to skin lesions is lower compared to the other more serious symptoms of COVID-19.

## Conclusions

We describe the incidence of skin involvement in patients who have contracted COVID-19 infection in Muscat Governorate in Oman. Skin manifestation of COVID-19 is not among the most commonly reported clinical presentation that alarm physicians to suspect the disease. We showed a low incidence of skin involvement in our sample compared to other reported incidence. However, at the time of the pandemic, unexplained skin lesions with or without other respiratory symptoms should alert physicians to consider COVID-19 as a cause. This will help minimize the spread of the disease. To our knowledge, this is the first report describing the skin manifestations of COVID-19-infected patients in Oman. More data that can represent the experience of other countries in the region and the pattern of skin involvement are needed.
